# Annotations

**Published:** 1893-02-25

**Authors:** 


### ANNOTATIONS.

**Dr. Tibbits' Libel Action.**

THE libel action of Tibbits v. Alabaster and Co. has ended in a verdict for the defendants, and Dr. Tibbits will, perhaps, now wish that he had left Mr. Harness and his belts alone or had spoken of them in more judicial and scientific terms. The defendants, the proprietors of the *Electrical Review*, appear to have come to the conclusion that Mr. Harness' belts are of little or no value, and that Dr. Tibbits' testimonial was of much the same quality as the belts. The bulk of the medical profession has long been of these opinions, and it is satisfactory to find that an independent authority like the *Electrical Review* has undertaken the task of exposure. The *Review* said some pretty strong things about both the belts and Dr. Tibbits, and it was supported in its general contentions by the greatest living authority on electrical questions, Lord Kelvin. According to the *Review* Dr. Tibbits, had exhibited "a most incredible ignorance of electrical laws," and the writer of the supposed libellous article had expressed the hope "that a grave scandal may at last be unveiled in all its grossness, and the promoters and abettors brought to book." These and similar statements constituted the alleged libel. "Are these things libellous?" asked the judge of the jury, or are they fit and proper things to be said? In about ten minutes the jury returned into court with the decision that the statements were not libellous, and that by consequence they were fit and proper things to be said. The verdict was, therefore, for the defendants, with costs. Dr. Tibbits will now probably consider that he has injured his scientific reputation for a very sorry "mess of pottage" indeed. As for Mr. Harness, he, doubtless, thinks that there are too many fools still left in the world for him to suffer in his pocket.

**A Blackburn "Hospital Scandal."**

COMPLAINTS have been made during the past year that the diet at the Blackburn and East Lancashire Infirmary has not been satisfactory to patients. Such complaints often get into newspapers under the heading of "More Hospital Scandals," and the like. We will ask the public to take note of the way in which the complaints have been dealt with in this particular case. We have it from headquarters that when the Matron first heard of complaints being made she inquired what they were and who had made them. They had been made by two visitors of the working-class, and they amounted to a wish on the part of some of the patients that the tea might be made a little sweeter. The matron thereupon sends to one of the wards and has a cup of the ordinary tea brought down for the inspection and experimental tasting of the two visitors. Both the visitors taste the tea, and one of them immediately declares that it is sweet enough, and the other that it is too sweet. So much for the sensible action of the matron. The committee, however, in order that there may be no "hushing up" of a scandal, make full inquiries into the subject, and find, as was authoritatively stated at the annual meeting the other day, that the complaints are "without foundation." Now this is such a manifestly trumpery affair that we should not confer upon it the gravity of print, but for one reason. The reason is that we are persuaded that it is a fair sample of the average "Hospital Scandal." The great majority of so-called "hospital scandals," we are fully convinced, have no more foundation than this. By way of justifying our contention, we give an outline of the "ordinary"—not the "generous," but the "ordinary"—daily diet of the patients at this very hospital. To begin with, five meals a day are provided for the patients. Breakfast consists of a pint of tea, with unlimited bread and butter. At lunch there is half-a-pint of milk or beef-tea. At the midday dinner five ounces of cooked meat are supplied to each patient, with half-a-pound of potatoes and half-a-pint of soup, or rice or bread pudding. For tea there is, as at breakfast, a pint of tea, with unlimited bread and butter; and for supper half-a-pint of milk or soup, or beef-tea if the latter is ordered. If this is not a sufficient dietary for sick persons in hospital, we would like to know what would be! How many hospital patients can command five such meals in their own homes every day? We happen to know also that the food is carefully cooked, that the meat and potatoes are sent to the table "piping hot," under covers, and that the covers are not removed until the plates are placed before the patients. The tea is always freshly made, and the bread and butter as carefully cut and prepared as if it were intended for a ladies' drawing-room. Perhaps one ought not to feel indignant. Blackburn abounds with working people, and gossipping mill hands of the female sex. And if a story is started in a "mill" among a thousand women and girls, one may understand the dimensions it will grow to before it has gone the round of thirty or forty such mills. We therefore excuse the mill-workers, as the Hospital Committee and officials have done. The people we cannot excuse are the newspaper writers, who, out of ridiculous materials like these, gravely construct for public use, "More Hospital Scandals."

**Doctors and the Drink Bill.**

THE modern doctor has long recognised the dangers of drink. If the *Times* may properly utter a lamentation over the Rev. Dr. Dawson Burns' presentation of the nation's annual drink bill THE HOSPITAL may turn the lamentation into a wail. The *Times* sees the evil effects of drink in the mass. THE HOSPITAL, including as it does all that appertains to medicine, sees them in all their painful, disastrous, and heartbreaking details. A vast sum of money is £140,886,262, and this enormous sum represents the spendings of the British Isles in alcoholic drinks during the past year. Probably three-fourths of this ocean of poison was swallowed by one-fourth of the population. We are not of those who stand for total abstinence, and therefore we may speak all the more freely. Doctors, all doctors, know now that alcohol is a powerful poison. In large doses it kills quickly man, woman, child, or beast; in small doses it kills more slowly, but not less surely. Alcohol of any considerable strength is not a fit substance to be used as a beverage, any more than an infusion of opium would be. It is not a proper substance for use as a regular food, except in its very weakest forms, and then not for all persons indiscriminately. If this be so, why have not doctors instituted a crusade against the abuse of alcohol, it may be asked? They have instituted such a crusade; and, considering that the abuse of alcohol brings many patients to the physician's consulting-room who would not otherwise be seen there, it is very much to their honour. If any testimony can be called disinterested on this subject, it is that of medical men; and medical men, if they had their way, would with one voice pronounce in favour of such a degree of moderation in alcohol as would be next door to total abstinence. And they would do this unhesitatingly for the whole population. There is not a responsible doctor anywhere but would say that if our national drink bill could be reduced from a hundred and forty millions to fourteen millions a year the health of the people would so improve that at least one fourth of our practising physicians would find nothing to do. As medical culture advances, so will medical influence grow; and as medical influence grows, the consumption of alcohol will diminish. If by any chance this should not be so, then the disregard of physiology thus evidenced will be as sure a sign of national decadence and degradation as the disregard of morals.

